# Efficacy and safety of Qushi Huayu granule for hyperlipidemia: study protocol for a randomized, double-blind, placebo-controlled trial

**DOI:** 10.1186/s13063-022-06031-3

**Published:** 2022-02-02

**Authors:** Yuanlong Sun, Na Hu, Gaofeng Chen, Yanjie Wang, Yiyang Hu, Maojun Ge, Yu Zhao

**Affiliations:** 1 Key Laboratory of Liver and Kidney Diseases (Ministry of Education), Institute of Liver Diseases, Shanghai Key Laboratory of Traditional Chinese Clinical Medicine, Shuguang Hospital Affiliated to Shanghai University of Traditional Chinese Medicine, No. 528 Zhangheng Road, Pudong New Area, Shanghai, 201203 China; 2 Institute of Clinical Pharmacology, Shanghai University of Traditional Chinese Medicine, Ministry of Education, Shanghai, 201203 China; 3 Department of Information Technology, Shuguang Hospital Affiliated to Shanghai University of Traditional Chinese Medicine, Shanghai, 201203 China

**Keywords:** Qushi Huayu granules, Hyperlipidemia, Traditional Chinese medicine, Randomized controlled trial

## Abstract

**Background:**

Hyperlipidemia has become a common chronic disease worldwide in recent years. Studies have shown that hyperlipidemia patients, especially those with a high level of serum low-density lipoprotein cholesterol (LDL-C), have a significantly higher prevalence of atherosclerosis, leading to coronary heart disease. Previous basic experiments and clinical studies have shown that Qushi Huayu granules (QSHY) reduce blood lipids in patients with non-alcoholic fatty liver disease (NAFLD) accompanied by hyperlipidemia. However, the clinical efficacy of QSHY in patients with hyperlipidemia is still lacking. This study aims to investigate the effect and safety of QSHY for hyperlipidemia.

**Methods:**

This is a randomized, double-blind, placebo-controlled trial. A total of 210 participants will be enrolled and randomized into the QSHY or placebo granules groups in equal proportions, who will receive treatment for 24 weeks. The primary outcome will be the change in LDL-C from baseline to week 12. Secondary outcomes will be changes in other serum lipids markers, life quality measuring health surveys, and traditional Chinese medicine (TCM) pattern scale. All related tests will be measured at baseline, week 12, and week 24 after enrollment. Adverse events and the safety of intervention will be monitored and evaluated.

**Discussion:**

We designed a clinical trial of hyperlipidemia management with QSHY, a TCM prescription. The results of this trial will present the efficacy and safety of QSHY in patients with hyperlipidemia.

**Trial registration:**

Chinese Clinical Trial Registry ChiCTR2000034125. Registered on June 25, 2019

## Administrative information

Note: the numbers in curly brackets in this protocol refer to SPIRIT checklist item numbers. The order of the items has been modified to group similar items (see http://www.equator-network.org/reporting-guidelines/spirit-2013-statement-defining-standard-protocol-items-for-clinical-trials/).

**Table:** 

Title {1}	Efficacy and safety of Qushi Huayu granule for hyperlipidemia study protocol of a randomized, double-blind, placebo-controlled trial
Trial registration {2a and 2b}.	Chinese Clinical Trial Registry, ID: ChiCTR2000034125. Registered on June 25, 2019.
Protocol version {3}	23 December 2019, version 2.0
Funding {4}	This study was funded by the Science and Technology Commission of Shanghai Municipality (19401970300).
Author details {5a}	(1) Yuanlong Sun, MD, PhD, E-mail: sunyuanlong@shutcm.edu.cn (2) Na Hu, MD, PhD, E-mail: 13994219533@163.com (3) Gaofeng Chen, E-mail: gaofengchen06@126.com (4) Yanjie Wang, E-mail: 1440327036@qq.com (5) Yiyang Hu, MD, PhD, E-mail: yyhuliver@163.com (6) Maojun Ge, PhD, E-mail: 0517@sgyy.cn (7) Yu Zhao, MD, PhD, E-mail: 3089@sgyy.cn
Name and contact information for the trial sponsor {5b}	Sponsor: Science and Technology Commission of Shanghai Municipality Coordinating Investigator (contact): Prof. Dr. Yu Zhao Institute of Liver Disease, Shuguang Hospital of Shanghai University of Traditional Chinese Medicine Zhangheng Road 528 Pudong New Area, Shanghai, China Email: 3089@sgyy.cn
Role of sponsor {5c}	The sponsor has no role in the design of the study; in the collection, analysis, and interpretation of the data; and in the writing of the manuscript.

## Background

Due to the change of lifestyle and diet, the incidence rate of hyperlipidemia has increased significantly [1]. Fifty-three percent of adults, or more than 100 million people in the USA, have elevated levels of serum LDL-C [2]. The prevalence of dyslipidemia in Chinese adults is as high as 40.40% [3], and 45% of the prevalence is in Russia [4]. Hyperlipidemia usually refers to elevated serum cholesterol and/or triglyceride [5] levels due to the disorder of systemic lipids metabolism. Among them, the increase of LDL-C is the main risk factor of atherosclerosis [6, 7] leading to coronary artery disease, myocardial infarction(MI), and stroke [8, 9]. In addition, studies showed the relationship between the control of blood lipids and the progress of diseases such as osteoporosis [10], chronic kidney disease [11, 12], acute pancreatitis [13].

The treatment of hyperlipidemia is based on lifestyle change therapy, which is generally required to improve the diet structure and lifestyle, but has little effect on blood lipids level when used alone [14], further, cooperate with Western medicine, and start drug treatment according to the risk of atherosclerotic cardiovascular disease (ASCVD) [15, 16]. At present, statins in clinical application are used as the cornerstone of lipid-lowering medicines [17]. Other drugs and therapies, such as fibrates, ezetimibe, proprotein convertase subtilisin/kexin type 9 (PCSK9) inhibitors, bile acid sequestrants, niacin, and gene therapies, including gene therapy of small interfering RNA, antisense oligonucleotides, viral vector-mediated, and non-coding RNA therapy [18–20], are still in the laboratory research stage or have not been widely used in clinical. In clinical, the lipid-lowering drugs represented by statins have certain limitations, the incidence of adverse reactions of statins is relatively high [21, 22]. The main adverse reactions are gastrointestinal reactions, thrombocytopenia [23], liver and kidney function damage, myopathy, hyperglycemia, and neurotoxicity [24]. Myopathy is the most common adverse reaction. Rhabdomyolysis occurred in severe cases [25], and even statin-induced necrotizing autoimmune myopathy (SINAM) [26]. With the in-depth study of related research, several studies suggest that statins have a risk of diabetes [27].

Traditional Chinese medicine (TCM) has a good effect on hyperlipidemia [28]. In the 2002 version of the “Guiding Principles for Clinical Research of New Chinese Medicines,” hyperlipidemia is classified as the “phlegm” category of TCM. Phlegm-blood stasis are the main pathological products of hyperlipidemia; the phlegm and blood stasis is a common syndrome type of hyperlipidemia [29]. Although TCM prescriptions or certain herbal active ingredients show the effect of lowering blood lipids in some existing pre-clinical research reports, there are currently only a few Chinese patented final products with clear lipid-lowering application indications, such as Xuezhikang capsules [30], Zhibituo [31], and Zhibitai capsules [32] are on the market. Therefore, the treatment of hyperlipidemia by TCM lacks evidence-based medicine data support and market transformation.

Qushi Huayu (QSHY) is an empirical prescription of TCM, which consists of five herbs. QSHY has been proven to alleviate liver steatosis and inflammatory damage in patients with non-alcoholic fatty liver disease (NAFLD) and obese rodent models [33–36]. QSHY treatment significantly reduced the elevation of serum LDL-C, TC, TG, ALT, and AST in NAFLD rodent models induced by HFD [35, 37], the related mechanism is that QSHY could regulate the dysbiosis of intestinal microbiota and inhibit hepatic lipogenesis [37, 38]. Although the experimental evidence indicates that QSHY could be used in the administration of hyperlipidemia, there is still a lack of evaluation of the effect of QSHY in the treatment of patients with hyperlipidemia compared with placebo. Therefore, this study will investigate the efficacy and safety of QSHY for hyperlipidemia.

## Methods

### Study design

**Fig. 1:**
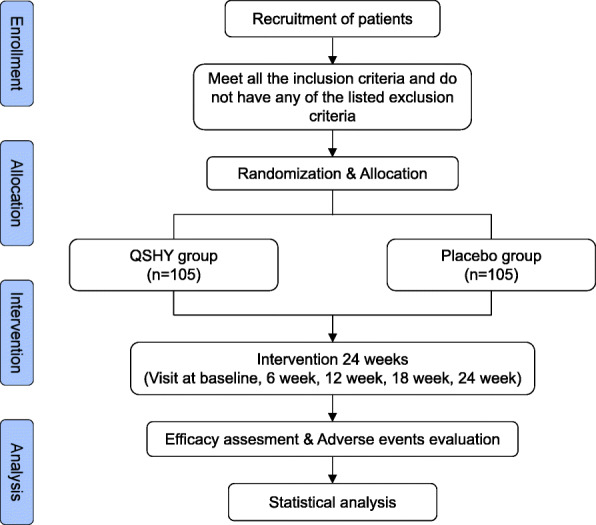
Flow diagram of this study protocol

This study will investigate the efficacy and safety of QSHY as a treatment for hyperlipidemia diagnosed with phlegm-blood stasis syndrome. This is a randomized, double-blind, placebo-controlled trial. The flow diagram of this trial is shown in Fig. 1. This study will be conducted in Shuguang Hospital Affiliated to Shanghai University of Traditional Chinese Medicine.

### Participants

#### Inclusion criteria

The following are the inclusion criteria:

1. For patients who meet the diagnostic criteria of Chinese and Western medicine for hyperlipidemia. The diagnostic criteria for hyperlipidemia refer to the Chinese Guidelines for the Prevention and Treatment of Dyslipidemia in Adults (2016 Revised Edition) [39]. TCM diagnosis, syndrome differentiation criteria, and their basis refer to the TCM diagnosis and treatment criteria for dyslipidemia formulated by the heart disease branch of the Chinese society of TCM [40].
2. Aged over 18 years.
3. Participates without ASCVD, 3.4 mmol/L < LDL-C < 4.9 mmol/L; ASCVD patients, 2.6 mmol/L < LDL-C < 4.9 mmol/L.
4. Patients with fasting TG < 5.7 mmol/L.
5. BMI < 35 kg/m^2^.
6. Willing and able to follow the scheduled visit plan, diet and exercise instructions, laboratory tests, and other procedures.
7. Patients who signed informed consent.

#### Exclusion criteria

The following are the exclusion criteria:

1. The patients who used other lipid-lowering drugs 4 weeks before enrollment
2. Patients with uncontrolled endocrine or metabolic diseases that may interfere with blood lipids levels
3. Patients taking oral corticosteroids
4. Patients with a history of alcohol or narcotic drug abuse within 1 year before screening
5. Patients with a high or very high risk of arteriosclerotic cardiovascular disease
6. Patients with congestive heart failure, unstable angina, MI, stroke, coronary artery bypass surgery or angioplasty, or unstable/serious peripheral artery disease in the past 6 months
7. Those who have undergone gastrointestinal surgery in the past year or those who have taken weight-loss drugs in the past 3 months and have lost more than 10% of their body weight
8. Pregnant or lactating female patients
9. Patients with serious primary diseases or other major diseases such as malignant tumors

### Randomization and allocation

The consecutive random sequence of 2 groups is provided by the clinical medicine center with SAS version 9.1 statistical software. Eligible participants will be randomly allocated into the QSHY group or the placebo group in a 1:1 ratio. The random sequence will be sealed in opaque envelopes and locked in the independent clinical medicine center. The allocation concealment will be maintained until all the data of each participant in each visit had been uploaded and locked.

### Blinding

#### Blind editing and blind bottom preservation

All patients and researchers will be blinded to the treatment assignments until the trial is completed. Personnel unrelated to this clinical trial will complete the preparation of drug blinding and emergency letters. Duplicate blinding is adopted in this study. All the data will be uploaded into the electronic database in double copies; the final statistical plan will be confirmed by question answering, verification, and blind review; and the database will be locked. After that, the first unblinding will be performed, and the results of the undefined group information corresponding to random numbers for necessary statistical analysis can be obtained. After the analysis, the main researcher will perform the second unblinding with the identification of two groups. All the unblinding processes will be recorded. In case of emergency, the reason, time, and place of blindness breaking will be recorded, and the cases will be treated as missing cases.

### Recruitment

Subject recruitment will be mainly carried out in outpatient clinics. Recruitment advertisement reviewed by the ethics committee will be performed in physical examination centers, outpatient halls, and other places where potential subjects gather to promote the recruitment. The investigators will be experienced physicians who are familiar with the trial process. A printed standardized protocol and an informed consent form will be presented during the investigation. To promote participant retention and complete follow-up, we will inform the subjects that their condition may improve after participating in this clinical study, and they can also obtain more medical advice and guidance related to this disease during the trial. Participants may also contribute to the research on the prevention and treatment of hyperlipidemia in TCM, which has social significance. The enrollment will be performed after the investigators fully introduce the detailed trial process, probable benefits, and potential risks to the eligible participant. The investigators will fulfill the case report form (CRF) after the participants have signed the informed consent form. All data collected from participants including personal information will be kept confidential in this trial.

### Interventions

Included participants in the treatment group will receive QSHY granules, while QSHY-simulated granules as placebo will be applied in the control group. Both groups of participants will receive regular health education, including diet control, exercise, and behavior modification. (1) Diet adjustment: diets with low sugar, low fat, and high vitamin will be recommended; (2) strengthen physical exercise: moderate metabolic equivalent exercise will be recommended for more than 30 min each time, 4 times a week; and (3) adjust emotion and keep a good mood.

The QSHY are compound preparations of 5 Chinese herbs, Artemisiae scopariae herba (Yin Chen), Polygoni cuspinati rhizome et radix (Hu Zhang), *Curcumae longae* rhizome (Jiang Huang), Gardeniae fructus (Zhi Zi), and Herba hyperici japonici (Tian Jihuang), packaged in consecutively numbered drug containers. The number was distributed according to the randomized number. The placebo will have an identically packaged appearance, weight, and taste compared to the QSHY granule. Both QSHY granule and placebos will be produced by Jiangyin Tianjiang Pharm Co. Ltd., Jiangsu, China. Participants will be advised to take one pack after meal, twice a day. The drugs are stored in the clinical trial base certified by Good Clinical Practice (GCP) and are managed and distributed in an independent clinical medicine center. The number of drugs received, taken, and returned by the patients will be recorded in detail by the research doctors at each visit. During the experimental period, non-pharmacologic care is permitted, but other TCM herbs and Western medication for lipid-lowering or fatty liver treatment will be prohibited.

Participants will be provided the information of the treatment and be advised not to take concomitant medications or change the dose and frequency of drugs autonomously. To facilitate completion, participants will be suggested to contact the arranged physicians for any questions raised about this trial. The drugs left over will be returned, and the related medication compliance will be calculated. Any combination of medications will be recorded in detail. Participants will be recommended to visit the outpatient clinic regularly for post-trial care.

### Study visit overview

**Fig. 2:**
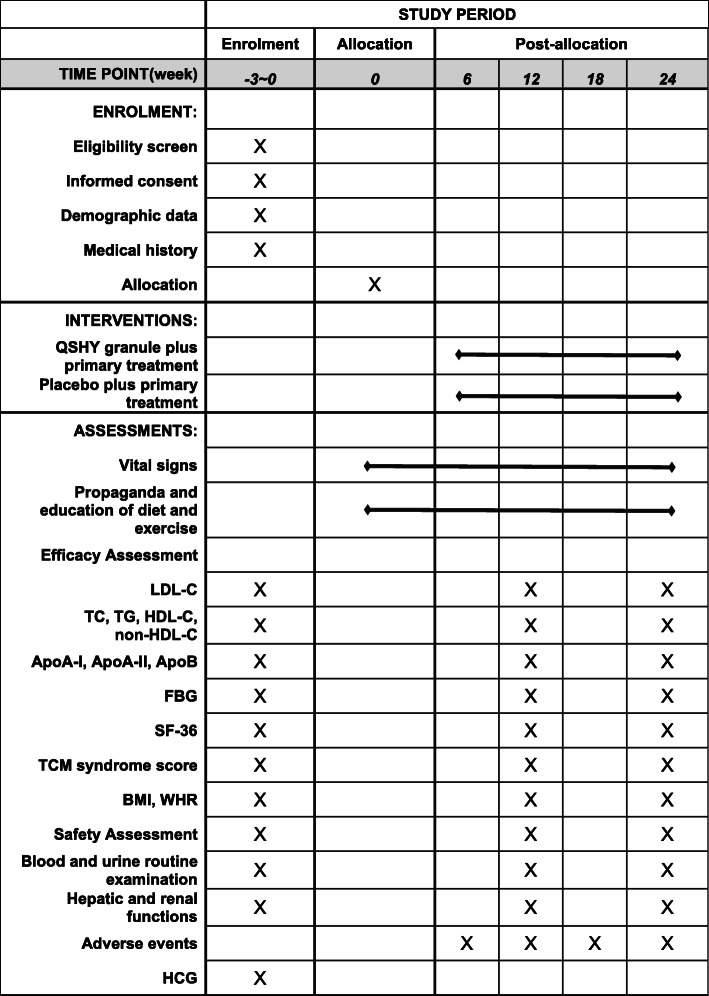
Study schedule of enrollment, interventions, and assessments. QSHY, Qu-Shi Hua-Yu granule; LDL-C, low-density lipoprotein cholesterol; TC, total cholesterol; TG, triglyceride; HDL-C, high-density liptein cholesterol; Apo, apolipoprotein; FBG, fasting blood glucose; SF-36, Medical Outcomes Study Item Short-Form Health Survey; TCM, traditional Chinese medicine; BMI, body mass index; WHR, waist-to-hip ratio; HCG, human chorionic gonadotropin

The visits in this trial are designed as 5-time points during 24 weeks of intervention: baseline, 6 weeks ± 5 days after enrollment, 12 weeks ± 5 days after enrollment, 18 weeks ± 5 days after enrollment, and 24 weeks ± 5 days after enrollment. At each visit time point, participants will be tested the laboratory index or recorded vital signs according to the study schedule. The detailed flow path information is shown in Fig. 2.

Blood samples will be collected from participants when they undergo blood examination and temporarily kept in evacuated and promoting coagulating tubes at 4 °C. After blood samples are centrifuged (4 °C, 3500 rpm/min, 10 min), serum samples will then be collected in Eppendorf tubes and stored orderly in refrigerators for clinical trials at − 80 °C.

### Adverse events process

If adverse events (AEs) occur, the researchers will judge its relationship with the trial, and take necessary measures to ensure the safety and rights of the participants. The severe adverse events (SAEs) should be reported to the IRB of Shuguang Hospital within 24 h. If moderate or severe adverse reactions judged to be related to the clinical trial occur in more than 25% of the total participants, this clinical trial will be terminated early.

### Outcomes

The primary outcome in this study is the change in LDL-C from baseline to week 12. The secondary outcomes are the changes in fasting serum TC, TG, HDL-C, non-HDL-C, apolipoprotein (Apo) A-I, ApoA-II, ApoB, and fasting blood glucose at 12-week assessment and 24-week assessment, and the change in the ratio of TC/HDL-C at 12-week assessment and 24-week assessment. All mentioned serum biochemical markers will be measured at baseline, 12-week, and 24-week after randomization. The measurement of life quality will be assessed by the Medical Outcomes Study Item short-Form Health Survey (SF-36) questionnaire. The changes in the syndrome of TCM for participants will be measured using four TCM diagnostic methods and the Scores of TCM syndrome Scale. Other outcomes are blood pressure, heart rate, body mass index (BMI), and waist-to-hip ratio (WHR).

Safety indexes are blood and urine routine examination and results of serum liver function test including AST, ALT, alkaline phosphatase (ALP), and serum renal function test including creatinine (CR) uric acid (UA) and blood urea nitrogen (BUN). Adverse events that occurred in the trial period will be monitored at each visit. All collected adverse events and their degree will be recorded with followed medical intervention in the CRF.

### Sample size

According to the previous results from a single-center clinical observation, after being treated with QSHY decoction, the fasting serum TC level decreased by 23.84% compared with baseline, and the fasting serum TC level in the control group decreased by 12.71% compared with baseline. It is expected that the decrease of fasting LDL-C level in the QSHY granule group will increase by 15% compared with placebo. Assuming *a* = 0.05, *β* = 0.2, *n*_*T*_: *n*_*C*_ = 1:1, *ẟ* = 0, *π*_*C*_ = 0.155, and *π*_*T*_ = 0.305, according to the formula of the optimal sample size, 94 samples in each group will be obtained. Considering the leakage rate of 10%, there will be at least 105 cases in each group.

$$ {n}_T={n}_C=\frac{{\left({z}_{1-\alpha }+{z}_{1-\beta}\right)}^2\left[{\pi}_T\left(1-{\pi}_T\right)+{\pi}_C\left(1-{\pi}_C\right)\right]}{{\left[\left({\pi}_T-{\pi}_C\right)-\delta \right]}^2} $$

### Data management

Data management will be applied by an electronic data capture system (EDC). Two data administrators will input and proofread the data independently. Divergences raised about the CRF and data proofread will be generated as a concentrator data ready queue (DRQ). Data administrators will send the DRQ to the researchers through the clinical supervisor. The researchers deal with the DRQ and return it as soon as possible. The data administrators will confirm the modification and enter the new data according to the responses of the researchers, and issue DRQ again if necessary. After reviewing and confirming the correctness of the database, the main researchers, applicants, and statistical analysts will lock the data. The locked data file will not be changed. Any problems found after data locking will be corrected. All research results (including personal data, laboratory test documents, CRF) appearing in the original medical records will be completely confidential to the extent permitted by law. The subject’s name will not appear in the CRF table, only the initials of the subject’s name and the number assigned when participating in the study. In relevant research summaries, articles, and public publications, if necessary, only the initials and number will appear.

### Statistical analysis

The statistical analysis will be performed by an independent statistician. After the trial protocol is determined, the statistician is responsible for formulating the detailed statistical analysis plan in consultation with the principal investigator. Statistical analysis will be conducted using the SAS version 9.1 statistical software.

The primary analyses for this study will adopt the intention-to-treat (ITT) principle. The full analysis set [41] includes participants who met the inclusion and exclusion criteria and received at least one evaluation after randomization. The per-protocol set (PPS) includes participants who had good compliance (80–120% of the trial drugs were used), did not take prohibited drugs during the trial, and completed the CRF. Safety analysis will be conducted based on a safety analysis set including participants who have received at least one intervention and safety evaluation.

Participants can withdraw at any time and will be recorded the reason for withdrawal. Participants who discontinue or deviate from intervention protocols will be informed by interviewers to get the reason for dropout. The original data of all withdrawn and terminated cases should be kept and recorded for further analysis. The last observation of participants who dropped out or existing missing data will be carried forward as the final data.

Continuous variables will be presented as mean ± standard deviation [42] or median with quartile range, according to the distribution. An independent *t*-test or the Mann-Whitney *U* test will be conducted to compare the differences between the groups. Dichotomous variables will be presented as frequency and percentage and analyzed with the chi-square test or Fisher’s exact test. Subgroup analyses will be performed based on groups with high heterogeneity if necessary.

### Ethics issue

The clinical trial protocol was jointly agreed upon by the main researcher and the sponsor and has been approved by the IRB of Shuguang Hospital affiliated with Shanghai University of TCM before implementation (ethics approval number: 2019-780-135-01). If the protocol is revised in the process of clinical trial implementation, it needs reapproval from the ethics committee. Researchers will be informed by meetings before another recruitment. Participants already included will be informed of these modifications by researchers of this trial with reserved contact information. This trial is funded and supervised by the Science and Technology Commission of Shanghai Municipality.

## Discussion

Hyperlipidemia is a companion to many metabolic diseases, such as NAFLD and coronary heart disease. According to previous studies, QSHY can improve liver function and blood lipids, including TC, LDL-C, and TG of HFD rodent models [34], and no adverse reactions were found [35, 37]. Three previous clinical trials in China [33, 34, 43] have shown that QSHY significantly reduces ALT and AST in patients with NAFLD and can reduce serum TG and TC, which indicates that QSHY has a therapeutic effect on patients with NAFLD and hyperlipidemia. However, the data presented are all based on the inclusion criteria of NAFLD patients, and precise data of QSHY for hyperlipidemia is still lacking. We designed this protocol to explore whether QSHY is an effective treatment of hyperlipidemia.

We designed a randomized, double-blind, placebo-controlled clinical trial based on a superiority framework. Hyperlipidemia can be improved by diet control and regular exercise. in this study, we include participants with mild to moderate abnormalities in LDL value and strengthened health education for the subjects. We excluded patients who must undergo lipid-lowering therapy, such as those who are at high or very high risk of ASCVD, or had unstable or severe arterial disease events 6 months before screening, to avoid violating ethical requirements. Therefore, we chose placebo as a comparator. The use of placebo control can clarify the absolute efficacy of the study drug and have higher efficiency.

A high level of LDL-C, not only a biomarker, appears to be a causal factor of the pathophysiology of ASCVD. According to the guidelines for the management of dyslipidemias issued by the European Society of Cardiology/European Atherosclerosis Society (ESC/EAS) in 2019 [44], serum LDL-C level is recommended as the main outcome for screening, diagnosis, and management of lipid modification to reduce cardiovascular risk. Thus, we set LDL-C, not TC or TG, as the primary outcome in this trial. According to the 2002 guiding principles of clinical research on new drugs of traditional Chinese medicine, participants are recruited according to the conventional TCM syndrome identification before randomization.

This protocol set up five visits after enrollment to supervise the 6 months of the treatment period. Observation items designed will be measured and recorded in the CRF. Participants will have propaganda and education on diet control and exercise. However, quantitative and accurate quality control cannot be achieved due to the diverse autonomy of patients, and the potential interference caused by lifestyle differences cannot be eliminated.

## Trial status

The final version of this protocol is 2.0 on 23 December 2019. Recruitment began on 23 June 2020 and will be completed on 23 December 2022. A total of 102 patients have been enrolled.

## Data Availability

The datasets used and/or analyzed during the current study are available from the corresponding author on reasonable request**.**
